# Conjugation of Proteins by Installing BIO-Orthogonally Reactive Groups at Their N-Termini

**DOI:** 10.1371/journal.pone.0046741

**Published:** 2012-10-08

**Authors:** Nagasundarapandian Soundrarajan, Sriram Sokalingam, Govindan Raghunathan, Nediljko Budisa, Hyun-Jong Paik, Tae Hyeon Yoo, Sun-Gu Lee

**Affiliations:** 1 Department of Polymer Science and Chemical Engineering, Pusan National University, Busan, South Korea; 2 Department of Chemistry, Technische Universität Berlin, Franklinstrasse, Berlin, Germany; 3 Department of Molecular Science and Technology, Ajou University, Suwon, South Korea; 4 Department of Applied Chemistry and Biological Engineering, Ajou University, Suwon, South Korea; Cardiff University, United Kingdom

## Abstract

N-terminal site-specific modification of a protein has many advantages over methods targeting internal positions, but it is not easy to install reactive groups onto a protein in an N-terminal specific manner. We here report a strategy to incorporate amino acid analogues specifically in the N-terminus of a protein *in vivo* and demonstrate it by preparing green fluorescent protein (GFP) having bio-orthogonally reactive groups at its N-terminus. In the first step, GFP was engineered to be a foldable, internal methionine-free sequence via the semi-rational mutagenesis of five internal methionine residues and the introduction of mutations for GFP folding enhancement. In the second step, the N-terminus of the engineered protein was modified *in vivo* with bio-orthogonally functional groups by reassigning functional methionine surrogates such as L-homopropargylglycine and L-azidohomoalanine into the first methionine codon of the engineered internal methionine-free GFP. The N-terminal specific incorporation of unnatural amino acids was confirmed by ESI-MS analysis and the incorporation did not affect significantly the specific activity, refolding rate and folding robustness of the protein. The two proteins which have alkyne or azide groups at their N-termini were conjugated each other by bio-orthogonal Cu(I)-catalyzed click chemistry. The strategy used in this study is expected to facilitate bio-conjugation applications of proteins such as N-terminal specific glycosylation, labeling of fluorescent dyes, and immobilization on solid surfaces.

## Introduction

The N-terminus of a protein is an attractive target site for functionalization to afford protein modifications such as PEGylation, glycosylation and fluorescent labeling; these modified proteins can be valuable sources for development of therapeutics and diagnostics [1]–[3]. Several chemical methods have been developed for N-terminal functionalization of a protein, but they are generally complicated and involve side reactions which yield heterogeneous products [4]–[6]. Biological methods for the N-terminal functionalization of a protein have recently been recognized as efficient ways to overcome the problems in chemical N-terminal modification. In particular, an approach based on the methionine (Met) residue substitution method allows the efficient production of proteins with an N-terminal specific functional group *in vivo* [7]–[9], which would pave the way to generate proteins with novel functions.

The Met residue substitution method introduces unnatural Met analogues into a protein by reassigning the Met codon globally in a protein sequence [5], [6]. The simple procedure using the Met auxotroph enables the production of a range of proteins with functional groups on a large scale. Bio-orthogonally reactive groups, such as L-homopropargylglycine (Hpg) and L-azidohomoalanine (Aha), have been incorporated into the Met positions of proteins *in vivo* by adding the Met surrogates instead of Met because the wild-type Met-tRNA synthetase recognizes the unnatural amino acids [5], [10]. In addition, engineering of the substrate specificity of Met-tRNA synthetase can expand the scope of this methodology [11], [12].

Bacterial proteins are synthesized from Met and the removal process of the start Met can be suppressed by selecting the second residue next to the Met carefully [7], [9]. Therefore, Met analogues can be incorporated into the N-termini of proteins using the Met residue substitution method. However, the presence of the internal Met codons in the target sequences limits the successful application of the Met residue substitution method for N-terminal specific functionalization due to the reassignment of unnatural Met surrogates to internal Met codons as well as to the first Met codon [7]–[9]. This problem can be overcome by engineering the protein sequence to be devoid of internal Met residues. Although this approach sometimes needs time-consuming protein engineering work to find internal Met-free variants having original functions of proteins, to our knowledge, this approach is the only one that makes the N-terminal specific modification of a protein possible.

Our previous report showed that a protein sequence could be engineered to be an internal Met-free using a consensus-based concept [8]. In the study, the internal Met residues of the single chain fragment variable (scFv) antibody sequence were replaced successfully with other conserved amino acids without affecting the activity of the protein. This allowed subsequent N-terminal specific functionalization of the scFv using the Met residue substitution method. The stability of scFv probably contributed to the success of the approach because stability of a protein is known to be related to the resistance to mutations [13], [14]. However, it is easily expected that the Met removal based on consensus sequences may not always work, because most proteins are marginally stable and thus cannot withstand multiple changes in their sequences [15]. In particular, hydrophobic residues such as Met are frequently located in the highly packed hydrophobic core, which makes it harder to generate functional Met-free protein sequences.

**Figure 1:**
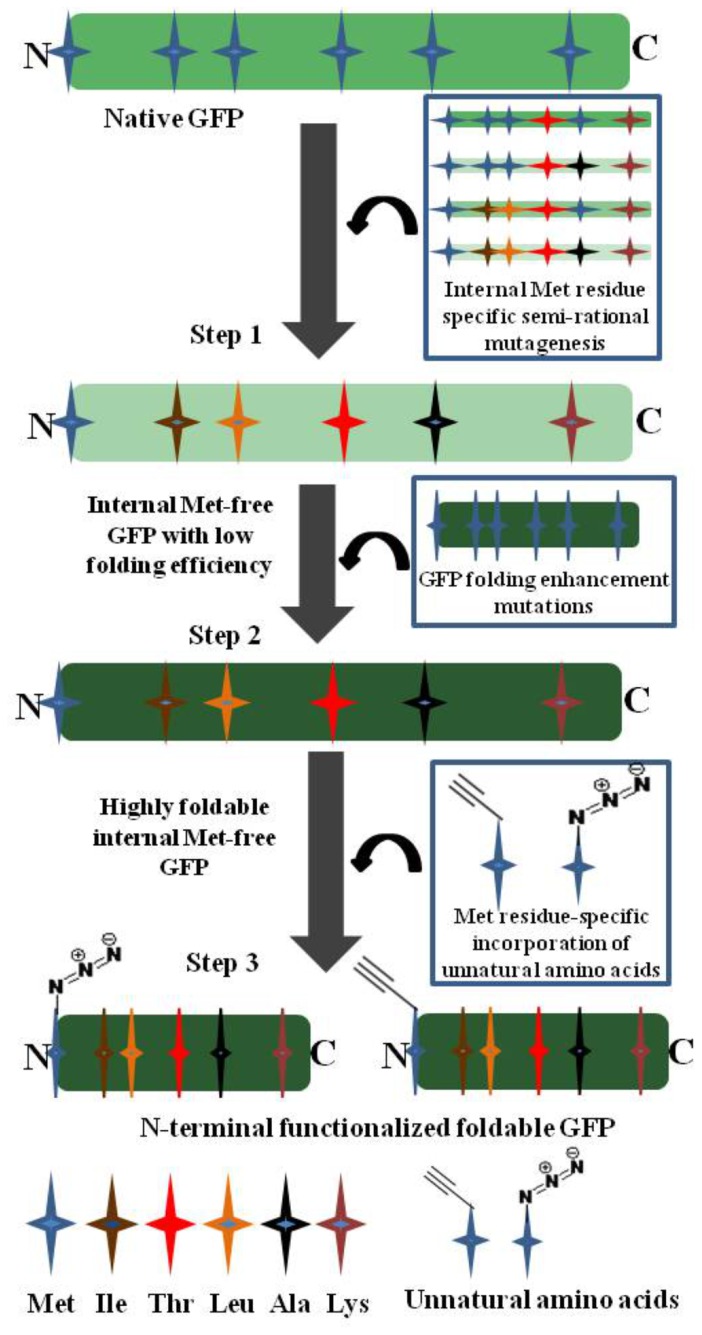
Schematic illustration of the approach for the generation of N-terminal functionalized green fluorescent protein (GFP). Step 1: generation of internal methionine (Met) free GFP by semi-rational mutagenesis using similar physicochemical amino acids. Step 2: introduction of folding enhancement mutations to rescue the fluorescence activity of internal Met-free native GFP. Step 3: N-terminal functionalization of active GFP by Met residue specific incorporation methodology.

We here engineered a green fluorescent protein (GFP) to be an internal Met-free protein sequence and demonstrated its N-terminal functionalization using the *in vivo* Met residue substitution method. It was previously reported that mutations of the three Met residues in the core hydrophobic regions of GFP based on consensus approach induced complete misfolding of the protein [16]. In the present study, a GFP devoid of internal Met residues was generated by semi-rational mutagenesis and its folding efficiency was improved by introducing mutations for GFP folding enhancement, which yielded an internal Met-free GFP sequence that can be properly folded. Subsequently, bio-orthogonally reactive amino acid analogues were introduced at the N-terminus of the engineered GFP (Figure 1). Then a protein-protein conjugation was demonstrated using the N-terminally modified GFPs.

## Materials and Methods

### Materials

T4 DNA ligase, restriction endonucleases and PCR reagents, were purchased from New England Biolabs (Tokyo, Japan). The isopropyl-D-thiogalactopyranoside (IPTG) and other chemicals were purchased from Sigma chemicals (St. Louis, MO, USA) unless otherwise indicated. Hpg and Aha were purchased from Chiralix (Nijmegen, Netherlands). *Escherichia coli* strain M15A methionine auxotroph was kindly donated by Prof. David A. Tirrell (Caltech, USA) and plasmid pQE80-L was obtained from Qiagen (Valencia, CA, USA).

### Construction of the GFP Variants

The pQE-80-GFP constructed in our previous study [17] was used for the template of GFP_nt_. Mutagenesis of the Met codons in GFP_nt_ were carried out by assembly PCR method [18]. Table S1 shows the primers used in the mutagenesis. The amplification of GFP_nt_ in pQE80-L using two sets of primers (GFP_nt_Out-F and GFP_nt_M153-R, and GFP_nt_M153-F and GFP_nt_OutR) was performed with VentR DNA Polymerase (New England Biolabs) to mutate M153 to Thr. The DNA fragments obtained from these PCR steps were purified on 1.5% agarose gels (Qiagen Gel Extraction Kit). Equimolar quantities of the fragments were used as the template for the assembly PCR using the following pairs of primers: GFP_nt_Out-F and GFP_nt_Out-R. Similarly, M233K mutation was introduced into GFP_nt_ containing M153T using two sets of primers (GFP_nt_Out-F and GFP_nt_M233-R, and GFP_nt_M233-F and GFP_nt_OutR) followed by assembly PCR described above, which generated GFP_nt_-r2M. GFP_nt_-r3M was obtained by the saturation mutagenesis of the M218 position in GFP_nt_-r2M using two sets of primers (GFP_nt_Out-F and GFP_nt_M218-R, and GFP_nt_M218-F and GFP_nt_OutR) and subsequent screening of fluorescent colonies. GFP_nt_-r4M was also obtained by the saturation mutagenesis of the M78 and M88 positions in GFP_nt_-r2M using respective sets of primers and subsequent screening of fluorescent colonies. GFPhs-r5M was commercially synthesized by Genescript Corporation (New Jersey, USA). The GFP_nt_-r2M, GFP_nt_-r3M, GFP_nt_-r4M and GFP_nt_-r5M were cloned into pQE80-L by using *Bam*HI and *Hind*III restriction sites, and GFPhs-r5M was cloned into the same plasmid by using *Eco*RI and *Hind*III sites. All of the constructs possessed hexa-histidine tags in their C-terminals.

### Expression and Purification of Met Analogues Incorporated GFP Variants

The GFP variants in pQE80-L was transformed into *E. coli* M15A Met auxotroph and expressed in minimal medium according to previously described protocols [14]. Briefly, the limiting concentration of Met (0.035 mM) allowed the cells to attain an OD_600_ 0.6–0.8 and the target proteins were induced with 1 mM IPTG followed by simultaneous addition of either Met or Met analogues (0.5 mM) and allowed the expression for 5 h. The harvested cells were suspended in lysis buffer (50 mM Na-Phosphate buffer pH 7.4 containing protease inhibitor) and disrupted by french press. The suspension was subsequently spun down to collect the soluble and insoluble protein fraction for SDS-PAGE analysis. The remaining soluble protein fractions were purified by Ni-NTA column chromatography (GE Healthcare Bio-Sciences, Sweden) by standard protocol. Elution fractions were analyzed by SDS-PAGE, and those that were enriched in the desired GFP variants were pooled and dialyzed against 1X phosphate buffered saline (PBS).

### Measurement of Fluorescence

Whole cell fluorescence assay was performed on cells with a 0.1 OD_600_, suspended in 1X PBS, by measuring fluorescence intensity by exciting at 485 nm and collecting emission at 515 nm with excitation/emission slits of 5.0 nm using Perkin Elmer/Wallac Victor 2 Multilabel Counter (1420-011). The protein samples were excited at 490 nm and emissions collected at 511 nm with excitation/emission slits of 5.0 nm were recorded on Hitachi FL spectrophotometer (F-4500) equipped with FL solution program for analysis of the spectra.

### Denaturation and Refolding of GFP Variants

Each purified GFP variant (30 µM) was unfolded at 95°C for 5 min in 1X PBS containing 8 M urea and 5 mM DTT. Refolding was carried out by 100-fold dilution of urea denatured samples at room temperature into 1X PBS containing 5 mM DTT. The concentrations of denatured proteins were adjusted to 0.3 µM and recovered fluorescence was measured using Hitachi FL spectrophotometer (490 nm excitation, 511 nm emission, 10 nm excitation/emission slit) for 30 min with an interval of 3 sec. The recovered fluorescence was normalized by dividing final fluorescence after 24 h value. The normalized values were fitted with Sigma Plot (Systat Software Inc., CA) using equations as described by previous report [19].

### Renaturation Equilibrium Measurement

Each purified GFP variant (100 µM) was denatured at 95°C for 5 min in 9 M urea in TNG buffer (25 mM Tris pH 7.5, 0.2 M NaCl, 5% Glycerol, 1 mM DTT). Equilibrium fluorescence values were measured by diluting the urea denatured proteins into refolding buffer (TNG) containing 5 mM DTT to various final concentration of urea (1–6 M), and allowing the refolding to proceed up to 24 h at 15°C. Fluorescence was measured by exciting at 485 nm and emission at 515 nm with excitation/emission slits of 5.0 nm and was recorded on Perkin Elmer/Wallac Victor 2 Multilabel Counter (1420-011). C_0.5_ was calculated by measuring the concentration of urea at which the 50% of initial fluorescence was recovered after 24 h of incubation, and the values were determined by sigmoidal fit using sigma plot (Systat Software Inc., CA) [19].

### Protein-protein Conjugation

Copper (I)-catalyzed cycloaddition reaction between Hpg incorporated GFPhs-r5M and Aha incorporated GFPhs-r5M was performed in a reaction mixture composed of 0.5 mg/ml of each protein (250 µl), 50 mM Tris.HCl pH 8 (100 µl), 50 mM CuSO_4_ (50 µl), 50 mM L-ascorbic acid (50 µl), and H_2_O (300 µl). Control was prepared in similar way without the addition of CuSO_4_ and L-ascorbic acid. The reaction mixture was shaken for 24 h at 4°C, and dialyzed against 1X PBS thrice at 4°C. Finally, the reaction mixtures were analyzed by SDS-PAGE.

## Results

### Structural Role of Methionine Residues in GFP

The wild-type GFP is a 27 kDa protein with a single chain polypeptide containing 238 amino acids. The protein folds into an 11 stranded β-barrel with a single α-helix running through the barrel to form a chromophore. The chromophore cyclization reaction begins once the GFP achieves its near-native like structure [20]. The fluorescent chromophore is directly correlated with the folding status of the protein, which makes GFP an excellent model protein for protein engineering study [21]. In the present study, GFP_mut3.1b_ with a fast folding property [22] bearing one N-terminal Met and five internal Met (M78, M88, M153, M218, and M233) was used as a native GFP variant, designated as GFP_nt_. Among the five internal Met residues, three Met residues (M78, M88, and M218) are located in the hydrophobic core of the protein and the remaining Met residues (M153 and M233) are exposed to solvent. The buried internal Met218 plays a major role in folding process by interacting with single Trp57 through sulfur-aromatic interactions, which is essential for the GFP folding process [23].

### Engineering of Internal Met-free GFP Sequences

**Figure 2:**
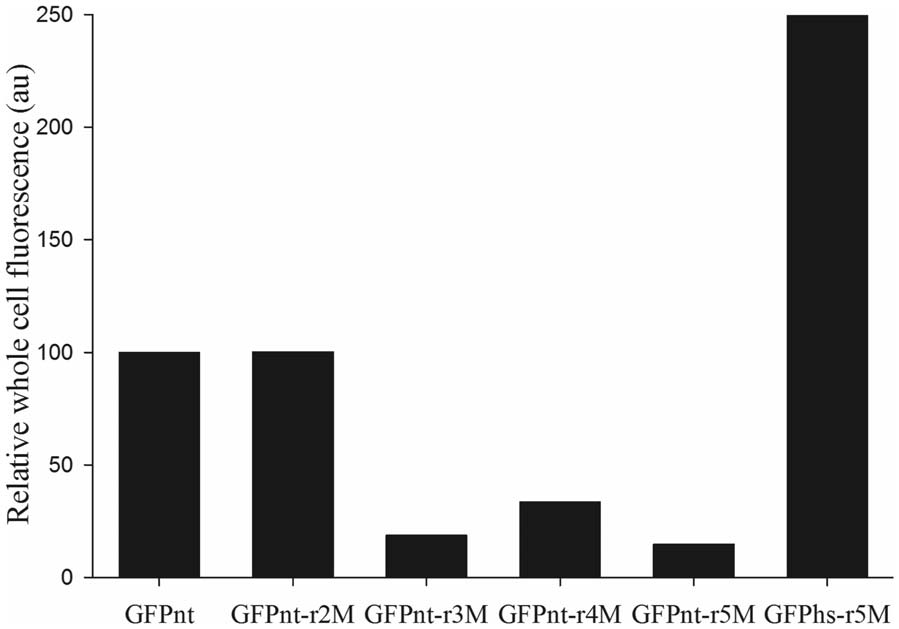
Comparison of functional productivity of GFP_nt_ and its variants. The data show the whole cell fluorescences of GFP_nt_ (containing all the 5 internal Met), GFP_nt_-r2M (containing M153T and M233K mutations), GFP_nt_-r3M (containing M153T, M233K and M218A mutations), GFP_nt_-r4M (M78I, M88L, M153T and M233K mutations), GFP_nt_-r5M (M78I, M88L, M153T, M218A and M233K mutation) and GFPhs-r5M (GFP_nt_-r5M containing mutations for folding enhancement). The relative fluorescence (in arbitrary units) is the fluorescence of whole cells compared with the fluorescence of cells expressing GFP_nt_.

To generate the internal Met-free GFP sequence, mutations of internal Met residues were attempted by considering the Met locations in the GFP structure. First, mutation of the surface exposed two Met residues (M153 & M233) in GFP was performed. Since the amino acid residues on the protein surface are relatively insensitive to mutations [24], [25], the two Met residues were mutated simply based on previous results instead of rigorous mutation studies. It was reported that the M153T mutation of GFP suppressed the aggregation of GFP [26]. For the M233 residue, our previous study informed that it was tolerable to the mutation of Lys [16]. Thus, the M153T and M233K variant of GFP_nt_, designated as GFP_nt_-r2M, was generated and the effect of mutation on the GFP activity and productivity were examined. GFP_nt_-r2M showed similar whole cell fluorescence (Figure 2) and soluble expression level (Figure S1A) with GFP_nt_, indicating that the mutations did not affect the GFP folding and activity significantly as expected.

As next step, we attempted to replace the remaining three internal Met residues (M78, M88, and M218) of GFP_nt_-r2M in the core hydrophobic region with other amino acids. Actually it was reported that the three Met residues can be mutated into other residues without affecting significantly the fluorescence of cells expressing the GFP variant (GFPrm_AM), in which M78, M88, and M218 were changed into Leu, Phe, and Ala respectively [12]. Therefore, we introduced the three mutations into GFP_nt_-r2M, but the fluorescence of cells expressing the GFP variant (M78L, M88F, M153T, M218A, and M233K) was much lower than that of cells expressing GFP_nt_-r2M. Since different GFP sequences were used as template to generate GFPrm_AM and the Met-free GFP in this study, we decided to change the three Met residues of GFP_nt_-r2M step by step. The M218 residue that plays an important role in folding [23] was randomized into hydrophobic amino acids (Leu, Ile, Val, Phe, and Ala) using oligonucleotides having degenerate codons. The clones showing fluorescence were selected manually based on their fluorescence, and a GFP_nt_-r2M variant having the M218A mutation, designated as GFP_nt_-r3M, showed the highest fluorescent among the mutants obtained. The whole cell fluorescence of the GFP_nt_-r3M was approximately 5 times lower than that of GFP_nt_-r2M (Figure 2). SDS-PAGE analysis confirmed that the reduced fluorescence of GFP_nt_-r3M was caused by a misfolding of the protein (Figure S1B), which highlights the importance of the M218 residue in the folding of GFP. Similarly, the other two internal Met positions (M78 and M88) in GFP_nt_-r2M were randomized at the same time with hydrophobic amino acids (Leu, Ile, Phe, Val, and Ala). A GFP_nt_-r2M variant having the M78I and M88L mutations, designated as GFP_nt_-r4M, showed the highest fluorescence; cells expressing GFP_nt_-r4M exhibited around 3-fold lower fluorescence than those expressing GFP_nt_-r2M (Figure 2). This result suggests that the M78 and M88 residues in the hydrophobic core are also important in GFP folding. All the three mutations, M78I, M88L, and M218A, were introduced into GFP_nt_-r2M, which resulted in a complete internal Met-free GFP sequence, GFP_nt_-r5M. However, the whole cell fluorescence of GFP_nt_-r5M was approximately 7 times lower than that of GFP_nt_-r2M (Figure 2), and GFP_nt_-r5M was mostly expressed as an insoluble form (Figure S1C). This confirms that the three Met residues in the hydrophobic core are very important in the formation of active GFP structure. Although it was not successful to generate an internal Met-free protein with preserved initial activity, these results suggest that the semi-rational approach based on similar physicochemical amino acids can be a handy tool for engineering a protein devoid of internal Met.

Both the three mutations M78L, M88F, and M218A in GFPrm_AM, and the mutations found in this study (M78I, M88L, and M218A) did not result in an active internal Met-free GFP variant. One thing that needs to be noted is that the starting GFP sequence to generate GFPrm_AM is a GFP variant (L024_3-3) that exhibited higher expression, better refolding behavior and higher stability than normal GFP [27], and thus we suspected that the properties of template GFP sequence could be an important factor for succeeding in generating an internal Met-free GFP variant. Since L024_3-3 was engineered to make GFP fluorescent with 5,5,5-trifluoroleucine, we turned to another GFP variant, superfolder GFP [19], which also showed improved folding properties and much more resistance to mutations than a wild type GFP. We introduced the mutations of superfolder GFP (S30R, Y39N, F64L, F99S, N105T, Y145F, M153T, V163A, I171V, and A206V) into GFP_nt_-r5M. It was also reported that N149K [28] and S208L [29] affected the folding efficiency of GFP positively, although their effects were not significant. The two mutations (N149K and S208L) were additionally introduced, and the resulting variant was named GFPhs-r5M. As shown in the Figure 2, the whole cell fluorescence of GFPhs-r5M was much higher than that of GFP_nt_-r5M, and approximately 2.5 times higher than GFP_nt_. SDS-PAGE analysis of the expressed protein confirmed that the soluble expression level of the GFPhs-r5M protein was improved significantly compared to that of GFP_nt_-r5M and higher than that of GFP_nt_ (Figure S1D), suggesting that the introduced mutations improved the folding efficiency of GFP_nt_-r5M remarkably. Table S2 shows the protein sequence of the soluble and active internal Met-free variant, i.e. GFPhs-r5M.

### N-terminal Functionalization of the Internal Met-free GFP

**Figure 3:**
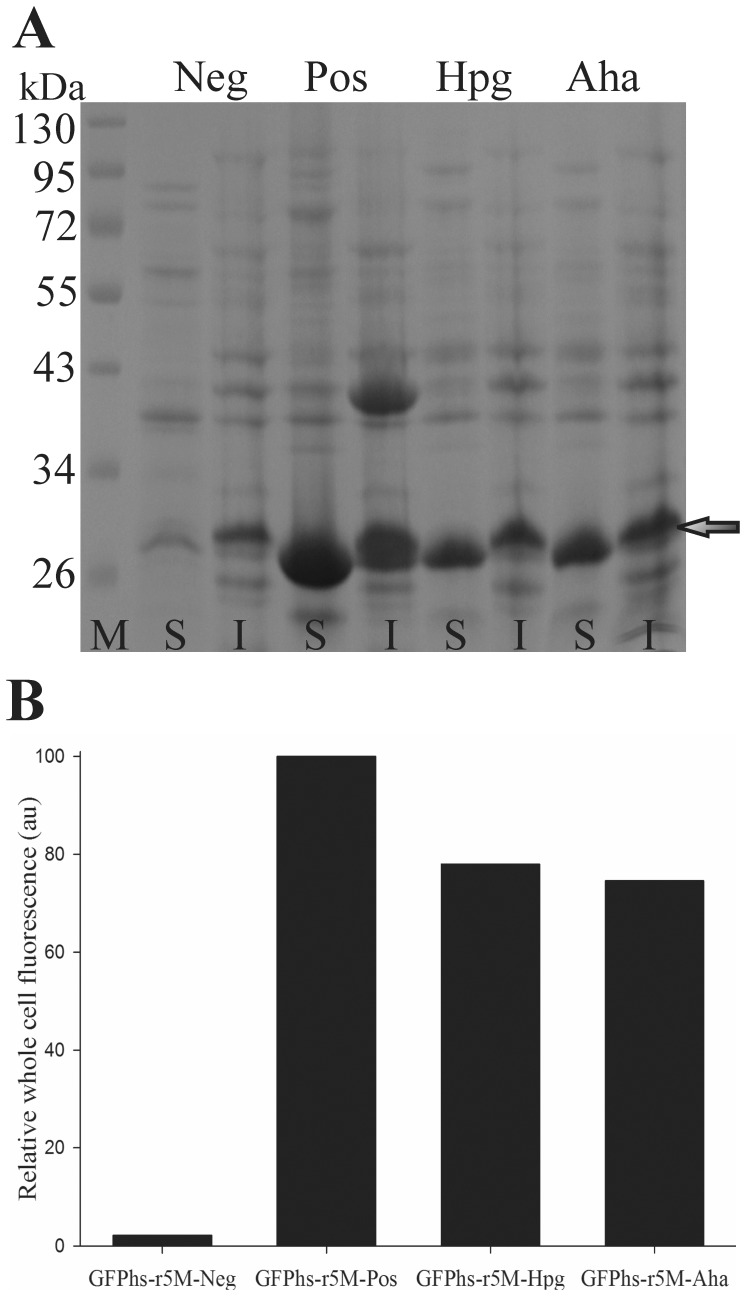
Effect of Met analogue incorporation on the productivity of active protein. (A) Protein expression profile of GFPhs-r5M incorporated with Met analogue in *E. coli* M15A. GFPhs-r5M was expressed without Met (GFPhs-r5M-Neg) or with Met (GFPhs-r5M-Pos) or with Met analogues such as Hpg (GFPhs-r5M-Hpg) and Aha (GFPhs-r5M-Aha) in minimal media containing 19 amino acids. The expected size is indicated by an arrow (S, soluble fraction; I, insoluble fraction; M, molecular weight marker). (B) Whole cell fluorescence of GFPhs-r5M expressed in minimal medium without Met (GFPhs-r5M-Neg) or with Met (GFPhs-r5M-Pos) or with Met analogues such as Hpg (GFPhs-r5M-Hpg) and Aha (GFPhs-r5M-Aha). The relative fluorescence (in arbitrary units) is the fluorescence of whole cells compared with the fluorescence of cells expressing GFPhs-r5M-Pos.

The GFPhs-r5M variant obtained from the above study is expressed as a functional form, and contains a Met residue only in its N-terminus, which suggests that the expression of the gene for GFPhs-r5M using the Met residue substitution method may enable the production of N-terminal functionalized GFP *in vivo*. To demonstrate this, the gene for GFPhs-r5M was expressed in the Met auxotrophic *E. coli* with the addition of Met surrogates, Hpg or Aha, according to the previously reported procedures [14]. Hpg and Aha are unnatural amino acids containing alkyne and azide groups respectively, which are illustrated in Figure S2. The soluble expression of GFPhs-r5M with Hpg or Aha was confirmed by SDS-PAGE (Figure 3A) and the corresponding active fluorescent proteins were produced despite an approximately 20% decrease in whole cell fluorescence compared to GFPhs-r5M with Met (Figure 3B). The proteins produced were purified and analyzed by ESI-MS to identify the incorporation of Hpg or Aha. The Hpg or Aha incorporated proteins showed an exact mass shift of −22 and −5 Da corresponding to one Met residue substitution of the respective unnatural amino acids (Figure S3). The ESI-MS data in the Figure S3 also showed an incorporation efficiency of >90%. These results clearly show that active GFP with N-terminal specific functional groups with high yield could be produced using the engineered GFPhs-r5M and Met residue substitution method.

### Characterization of the Functionalized GFP Variants

The specific fluorescence, refolding rate and folding robustness of the N-terminal functionalized GFPhs-r5M with Hpg or Aha (designated as GFPhs-r5M-Hpg and GFPhs-r5M-Aha respectively) were compared with those of GFPhs-r5M to examine the biophysical effects of N-terminal functionalization on the protein. The biophysical properties of GFP_nt_ were also examined and compared as a control.

**Figure 4:**
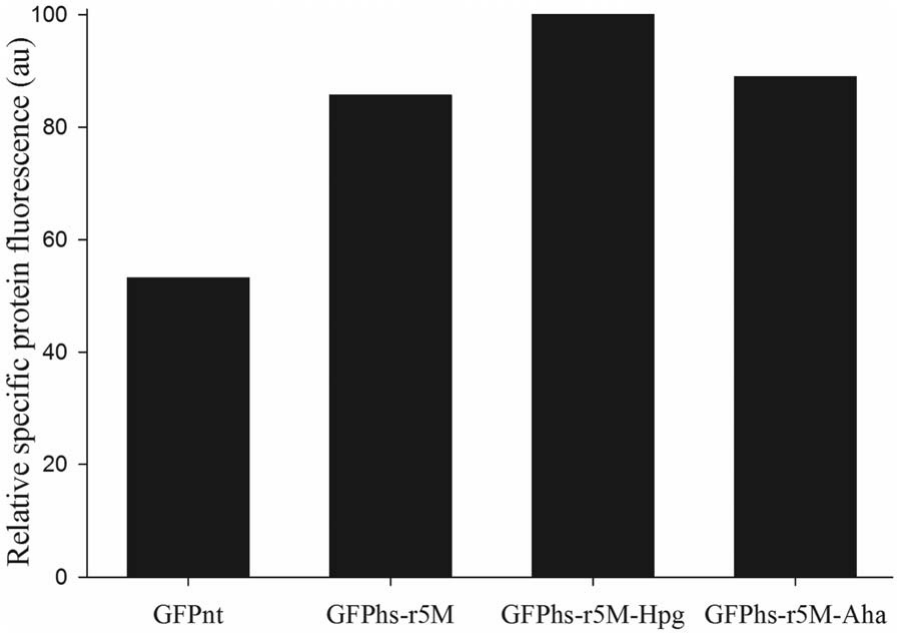
Relative specific fluorescene of GFP_nt_, GFPhs-r5M, GFPhs-r5M-Hpg and GFPhs-r5M-Aha of purified proteins. Relative specific activity (in arbitrary units) is the fluorescence of purified protein compared with the fluorescence of purified GFPhs-r5M-Hpg.

As shown in Figure 4, GFPhs-r5M, GFPhs-r5M-Hpg and GFPhs-r5M-Aha exhibited similar specific fluorescence activities, which suggest that the addition of alkyne or azide on the N-terminus of the protein did not affect the protein activity negatively. On the other hand, the specific fluorescent activities of GFPhs-r5M and its derivatives were approximately 1.5–2 fold higher than that of GFP_nt_. This indicates that the mutations introduced into GFP_nt_-r5M for folding enhancement had influence on the spectral properties of protein in addition to the folding efficiency. This also suggests that the higher whole cell fluorescence of GFPhs-r5M than that of GFP_nt_ in Figure 2 was caused by an enhancement of the specific fluorescent activity as well as by an increase in the soluble expression level.

**Figure 5:**
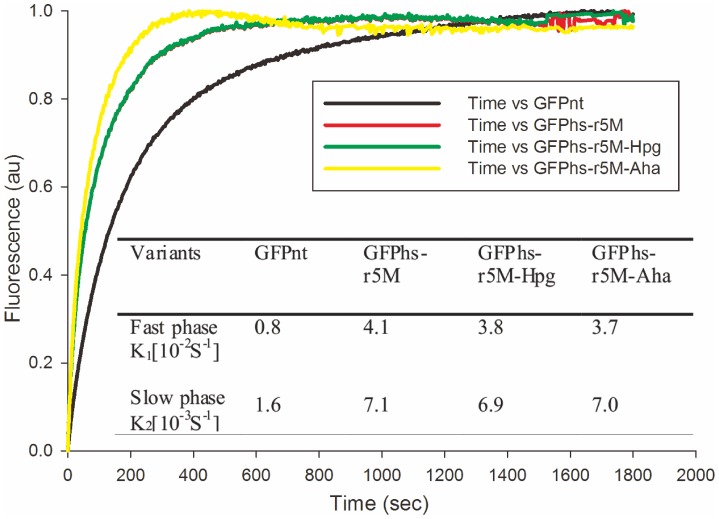
Refolding kinetics of the variants GFP_nt_, GFPhs-r5M, GFPhs-r5M-Hpg and GFPhs-r5M-Aha. Refolding kinetics was measured after denaturation in 8M urea followed by renaturation by dilution. Inlet table shows the refolding rates of GFP_nt_ and GFPhs-r5M variants for fast and slow phase of refolding progress. Normalized fluorescence in arbitrary units was plotted against time.

Figure 5 shows the refolding kinetics of the GFP_nt_, GFPhs-r5M, and GFPhs-r5M with Hpg or Aha. Both GFPhs-r5M-Hpg and GFPhs-r5M-Aha showed similar folding rates in both the fast and slow phases compared to GFPhs-r5M, which were 4–5 fold higher folding rate compared to GFP_nt_. These results are correlated with the soluble expressions level of GFP_nt_ and GFPhs-r5M (Figure S1).

**Figure 6:**
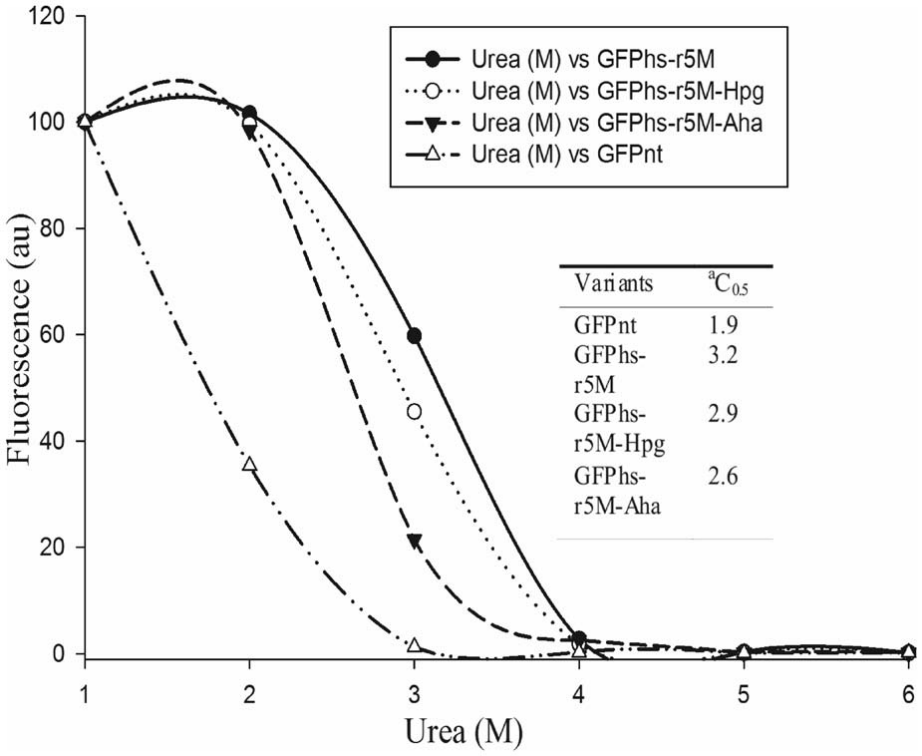
Equilibrium renaturation plots of GFP_nt_ and GFPhs-r5M variants (fraction of recovered fluorescence). Urea-denatured protein samples were diluted in different concentrations of urea in refolding buffer and recovered fluorescence was normalized by dividing it by fluorescence of corresponding non-denatured samples diluted in similar fashion. Inlet table shows the ^a^concentration of urea at which the 50% of fluorescence is recovered during renaturation process under urea-unfolded conditions.

The study on folding robustness was carried out by estimating the refolding tolerance of the four GFP variants to protein denaturant. The fractions of recovered fluorescence under different urea concentrations were determined after 24 hours and their C_0.5_ were estimated from the refolding equilibrium plot (Figure 6). The estimated C_0.5_ values of the GFP variants suggest that the incorporation of the unnatural amino acids has little effect on the folding robustness.

Overall, the GFPhs-r5M and its variants with N-terminal specific functional groups showed comparable biophysical properties, and their specific activity, refolding rate and folding robustness were higher than GFP_nt_. This suggests that the functionalized unnatural proteins produced were active and stable enough for further study such as bio-conjugation through the introduced functional groups. The results also support the possibility that the introduced mutations for GFP folding enhancement provided the GFP sequence with sufficient folding robustness to withstand the cumulative effects of internal Met-free mutations.

### Protein-protein Conjugation Using the Functionalized Proteins

Azides and alkynes are highly energetic functional groups with a particularly narrow distribution of reactivity. In addition, the copper (I) catalyzed cycloaddition reaction of azide and alkyne yields 1,4-disubstituted 1,2,3-triazole linked conjugates under very mild conditions such as room temperature and in an aqueous buffer. Moreover, the reaction is highly regiospecific, chemoselective and tolerant to a wide range of functional groups [1], [30], [31]. These outstanding features of click chemistry have been extended to various bio-conjugation applications.

**Figure 7:**
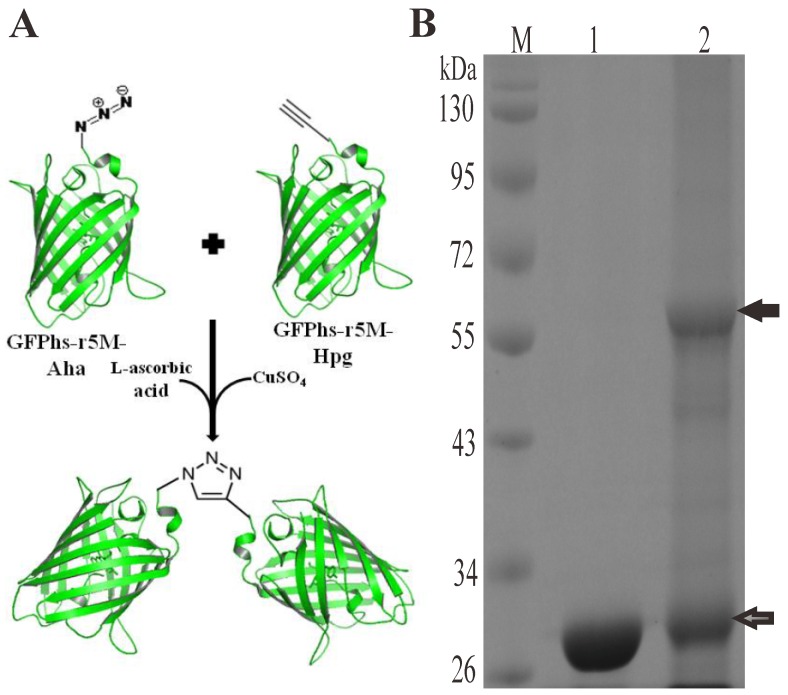
Protein-protein bio-conjugation of GFPhs-r5M-Hpg and GFPhs-r5M-Aha. (A) Copper (I)-catalyzed cycloaddition (CCCA) reaction between azide and alkyne incorporated to GFPhs-r5M resulted in the formation of triazole-linked protein-protein dimer bio-conjugation. (B) SDS-PAGE analysis of CCCA reaction between GFPhs-r5M proteins incorporated with Hpg (alkyne) and Aha (azide group). Lane 1: CCCA reaction without catalysis agents, CuSO_4_ and L-ascorbic acid; lane 2: CCCA reaction with catalysis agents, CuSO_4_ and L-ascorbic acid. This result shows the formation of triazole-linked protein-protein bio-conjugation of GFPhs-r5M dimer. M is molecular weight marker, thick arrow indicates the protein-protein conjugated GFPhs-r5M dimer of 55.2 kDa and grey arrow indicates the 27.6 kDa monomer of GFPhs-r5M containing Hpg and Aha respectively.

Protein-protein bio-conjugation reaction based on the click chemistry described above was performed to evaluate the possibility of bio-conjugation using the N-terminal specific functionalized GFPs produced *in vivo*. The purified GFPhs-r5M-Hpg and GFPhs-r5M-Aha containing an alkyne and azide groups, respectively, on their N-termini were incubated in the presence of CuSO_4_ and L-ascorbic acid to carry out the cycloaddition (Figure 7A). The bio-conjugation was analyzed by SDS-PAGE. Formation of the GFPhs-r5M dimer, 55 kDa in size, was observed with a yield of approximately 50% (Figure 7B), whereas the control reaction performed without CuSO_4_ and L-ascorbic acid did not produce such dimer band. This suggests that the protein-protein conjugation between the N-terminal specific functionalized proteins was achieved in a site-specific manner. In the protein-protein conjugation reaction, we could observe some protein aggregation and this hampered further characterization of the conjugated protein. This problem should be solved to use the protein-protein conjugation method more efficiently.

## Discussion

This study has demonstrated the biological N-terminal site-specific modification of GFP with bio-orthogonally reactive groups and its application to conjugation of the protein. The preparation of GFPs with N-terminal specific functional groups could be achieved by generating the internal Met-free GFP sequence that can be properly folded and subsequent use of the Met residue-specific substitution method. The produced unnatural GFPs were sufficiently homogeneous and showed almost comparable activity and folding efficiency to the natural GFP. Finally, the protein-protein conjugation using the functionalized GFPs was successfully demonstrated. Even though it was not done in this study, the N-terminally functionalized GFPs, GFPhs-r5M-Hpg or GFPhs-r5M-Aha, can be conjugated to the N-terminally functionalized scFvs, scFv-Aha or scFc-Hpg, which were prepared in our previous study [8], and the resulting GFP-scFv conjugates are fluorobodies which can be used for developing biosensors and diagnostics [32]. It is expected that the strategy employed in our study may not only enable the preparation of artificial N-terminal functionalized proteins on a large scale but also facilitate the various protein related bio-conjugation studies such as N-terminal specific glycosylation, PEGylation and labeling.

Although *in vivo* site-specific incorporations of unnatural amino acids into target proteins using stop codons have been successfully achieved by suppressor based methodology [33], this approach has not been demonstrated for the N-terminus of proteins. The Met residue-specific methodology used in this study is probably the only method to install amino acid analogues in the N-terminus of a protein, and this approach is a handy choice for N-terminal specific functionalization of target proteins when the internal Met residues of the proteins can be changed into other ones. Sometimes the internal Met residues of proteins could be replaced with other hydrophobic amino acids such as Leu, Ile, and Ala [7], [8]. However, it is easily expected that Met residues could play an important role in folding and stability of proteins, and these Met residues cannot be mutated to other ones without hurting the function or structure of the proteins. One of the key points in our study is that even the protein sequences such as GFP, of which the Met residues are very sensitive to mutations, could be engineered to be an internal Met-free sequence by introducing mutations known to enhance the folding efficiency and robustness of the protein. Although the mutations previously identified were used for the enhancement of GFP folding and stability in this study, current protein engineering approaches such as directed evolution and computational protein engineering can be efficiently employed in the identification of such folding enhancement mutations for other proteins [24]. This implies that the generation of the internal Met-free sequences which can be properly folded may not be a serious problem anymore in the preparation of the N-terminal functionalized proteins through the *in vivo* Met-residue specific substitution method. This also indicates that it is possible to artificially manipulate the incorporation sites of target proteins by genetically reassigning the Met codons to any sites of the internal Met-free protein sequence, which would allow the selective site-specific functionalization of a protein. In the case that the unnatural amino acids incorporated into the first Met codon is not required, it can be removed by engineering the penultimate residue with non-bulky amino acids such as Gly, Ala, Cys [7], [9], [34].

There are some general or specific limitations in the proposed method, which should be considered before applying the method to bio-conjugations. For example, the method may be very inefficient for the proteins with N-terminal signal sequences which can be cleaved *in vivo* or with hidden N-termini where the incorporated non-natural amino acids cannot be accessed once incorporated. In addition, the target proteins need to be purified to execute highly specific bio-conjugation reactions because the unnatural amino acids can also be slightly incorporated into endogenous proteins.

In our study, the mutations of the Met residues in the buried hydrophobic core regions of GFP significantly lowered the folding efficiency of GFP, which was rescued by introducing the mutations for GFP folding enhancement, the majority of which were from the superfolder GFP [19]. According to the structural analysis of the superfolder GFP, the mutations resulted in the higher folding rate and folding robustness by inducing new noncovalent interactions involving ionized residues [19]. For instance, the S30R mutation contributed the formation of double salt bridges with E17 and E32 and intramolecular ionic network through four residues (E17, E32, R122 and E115) located in four different adjacent β-sheets in the structure. It is presumed that this kind of superfolder mutation effect compensated the destabilization effect caused by the mutations of the three Met residues in the hydrophobic-core [19]. The higher folding efficiency and folding robustness of GFPhs-r5M than those of GFP_nt_ indicates that the superfolder mutations might presumably provide GFP_nt_-r5M with more stabilization energy than such compensating energy. On the other hand, we presume that the higher specific fluorescence of GFPhs-r5M than GFP_nt_ might be caused by the mutations such as F64L, F99S and N149K mutations which can change the spectral properties of GFP by enhancing the hydrogen bonding networks around the chromophore [22], [26], [28], [29]. Further mutagenesis and structural studies need to be performed to understand the improved folding and spectral properties of the variants more exactly.

## Supporting Information

Figure S1 A. SDS-PAGE analysis of the soluble and insoluble protein fractions of GFPnt and GFPnt-r2M. (M: molecular weight marker, lane 1: insoluble fraction of GFPnt, lane 2: soluble fraction of GFPnt, lane 3: soluble fraction of GFPhs-r2M, lane 4: insoluble fraction of GFPhs-r2M) B. SDS-PAGE analysis of the soluble and insoluble protein fractions of GFPnt-r3M. (S, soluble fraction; I, insoluble fraction). C. SDS-PAGE analysis of the soluble and insoluble protein fractions of GFPnt-r5M. (S, soluble fraction; I, insoluble fraction). D. SDS-PAGE analysis of the soluble and insoluble protein fractions of GFPhs-r5M and GFPnt. (S, soluble fraction; I, insoluble fraction; M, molecular weight marker). The expected size of 27.6 kDa is indicated by arrow in the figures.
(TIF)

Figure S2 **Chemical structure of natural L-methionine (Met) and their unnatural surrogates L-homopropargylglycine (Hpg) and L-azidohomoalanine (Aha) (Mol. Wt: molecular weight).**
(TIF)

Figure S3 **ESI-MS analysis of GFPhs-r5M incorporated with Hpg and Aha. GFPhs-r5M (A), GFPhs-r5M-Hpg (B) and GFPhs-r5M-Aha (C) incorporated with Met, Hpg and Aha, respectively.** *Inset* table of each spectra shows calculated and found masses. The peaks corresponding to found masses with Met, Hpg and Aha incorporated proteins might be due to cleavage of 8 residues. We generally could observe these peaks with almost all of the samples of GFPhs-r5M variants. The GFPhs-r5M containing Hpg and Aha showed the mass shift of −22 and −5 Da respectively, compared to GFPhs-r5M with Met.
(TIF)

Table S1 **Oligonucleotides used for saturation mutagenesis of internal Met-free GFP construction.**
(TIF)

Table S2 **Amino acid sequence of the GFPhs-r5M.** Red indicates Met replacement mutations, and green indicates the mutations for folding enhancement. The variant expressed as recombinant protein contains a hexahistidine tag sequence in the C-terminus of the protein for Ni-NTA purification.
(TIF)
