# Supplementary figures and images for: Conjugation of Proteins by Installing BIO-Orthogonally Reactive Groups at Their N-Termini
Source: PLoS One. 2012 Oct 8;7(10):e46741. doi: 10.1371/journal.pone.0046741 (PMC3466299; doi:10.1371/journal.pone.0046741)

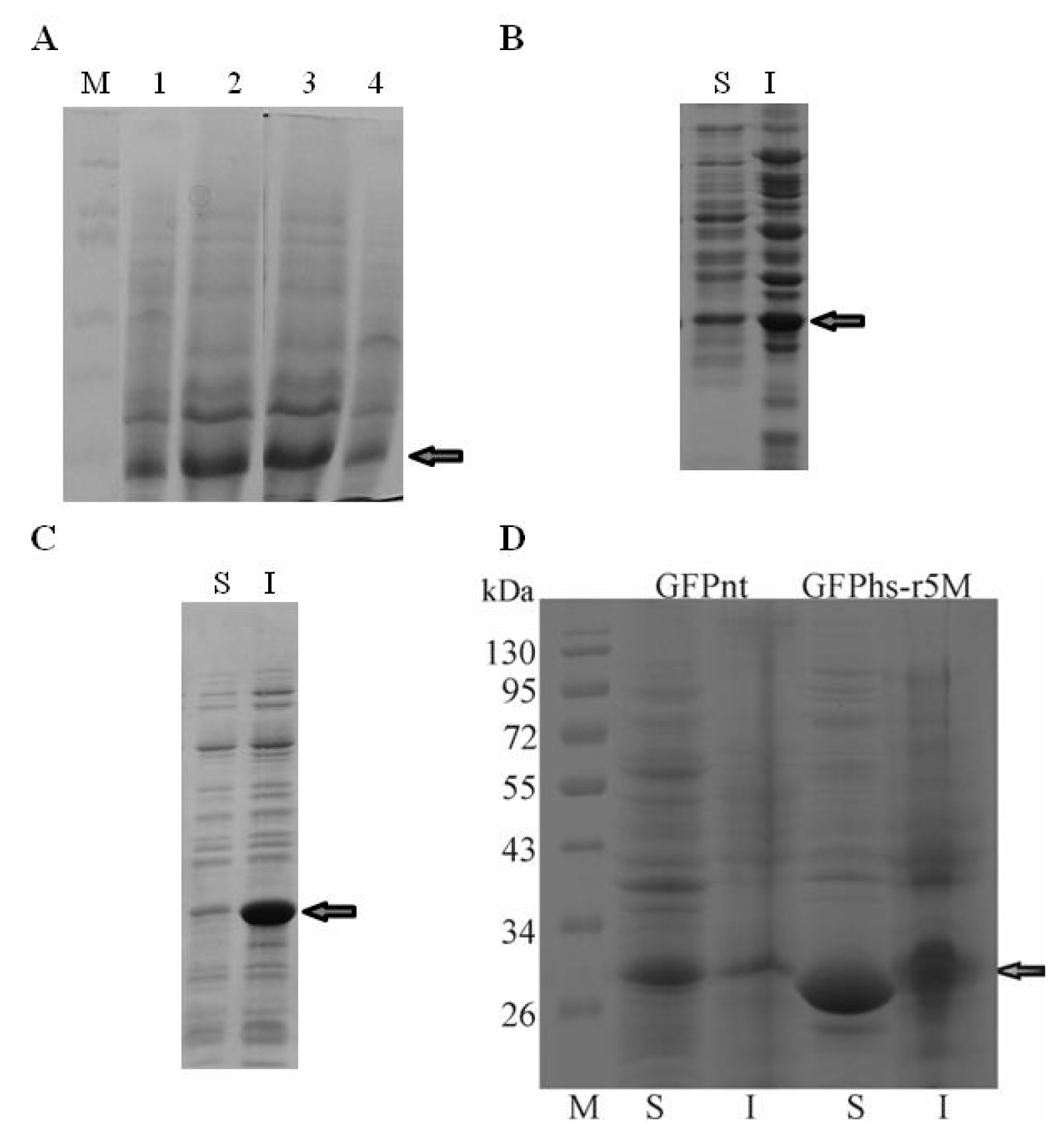

Supplement: Figure S1 — A. SDS-PAGE analysis of the soluble and insoluble protein fractions of GFPnt and GFPnt-r2M. (M: molecular weight marker, lane 1: insoluble fraction of GFPnt, lane 2: soluble fraction of GFPnt, lane 3: soluble fraction of GFPhs-r2M, lane 4: insoluble fraction of GFPhs-r2M) B. SDS-PAGE analysis of the soluble and insoluble protein fractions of GFPnt-r3M. (S, soluble fraction; I, insoluble fraction). C. SDS-PAGE analysis of the soluble and insoluble protein fractions of GFPnt-r5M. (S, soluble fraction; I, insoluble fraction). D. SDS-PAGE analysis of the soluble and insoluble protein fractions of GFPhs-r5M and GFPnt. (S, soluble fraction; I, insoluble fraction; M, molecular weight marker). The expected size of 27.6 kDa is indicated by arrow in the figures. (TIF) [file pone.0046741.s001.tif]

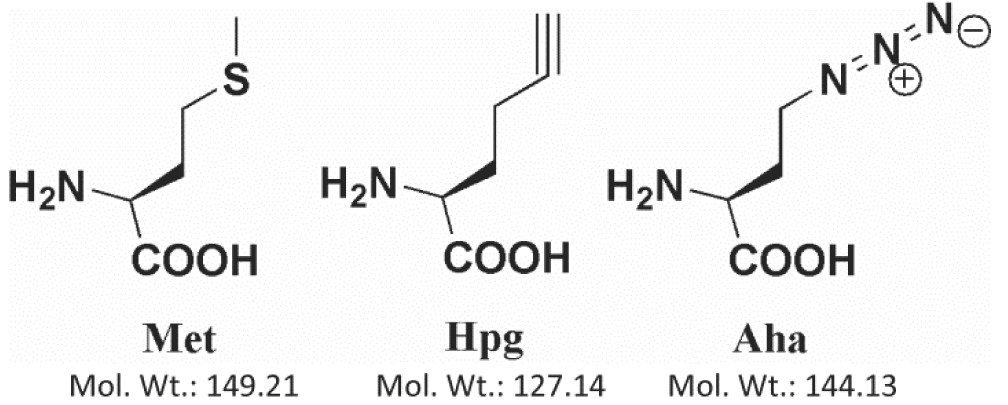

Supplement: Figure S2 — Chemical structure of natural L-methionine (Met) and their unnatural surrogates L-homopropargylglycine (Hpg) and L-azidohomoalanine (Aha) (Mol. Wt: molecular weight). (TIF) [file pone.0046741.s002.tif]

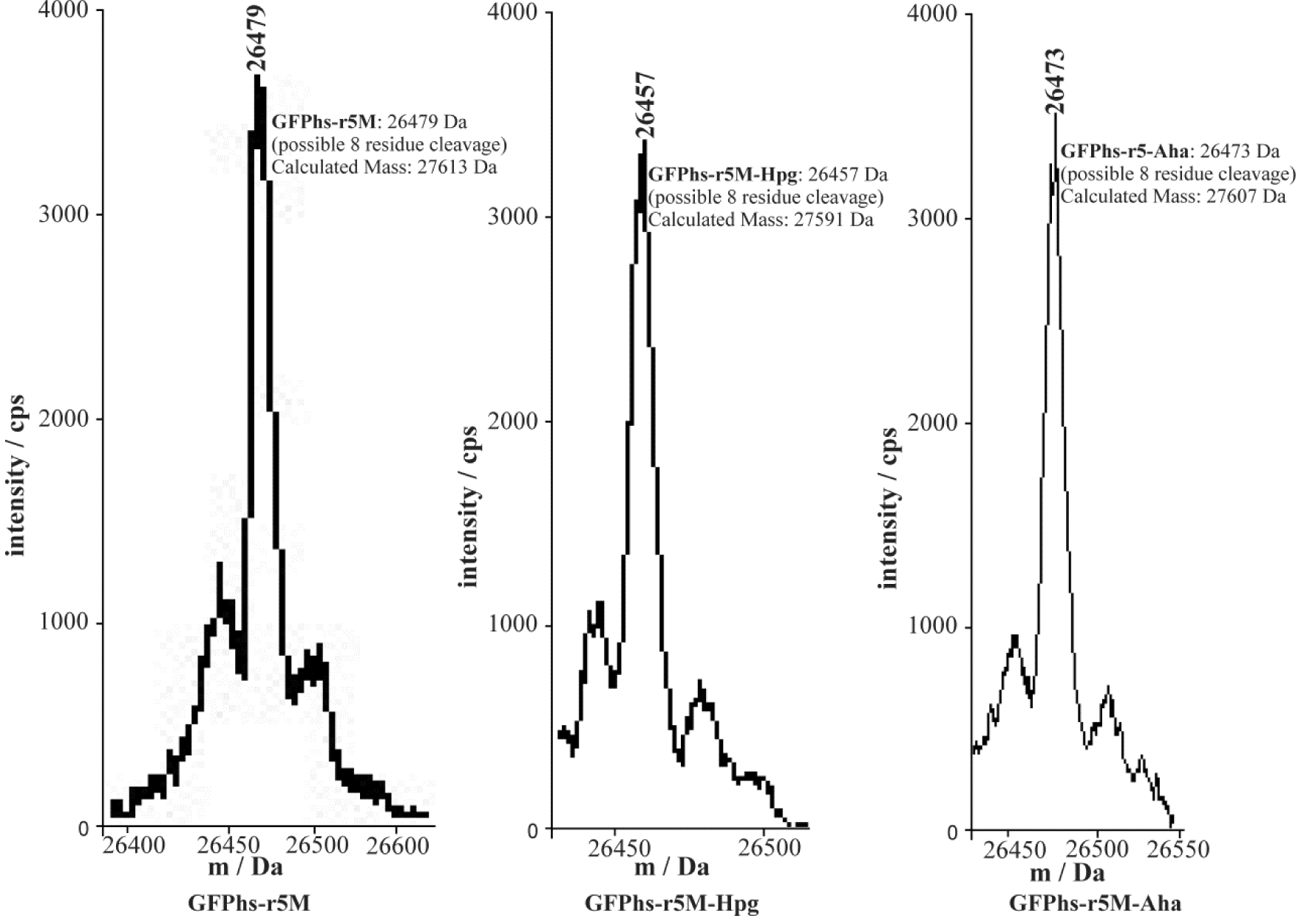

Supplement: Figure S3 — ESI-MS analysis of GFPhs-r5M incorporated with Hpg and Aha. GFPhs-r5M (A), GFPhs-r5M-Hpg (B) and GFPhs-r5M-Aha (C) incorporated with Met, Hpg and Aha, respectively. Inset table of each spectra shows calculated and found masses. The peaks corresponding to found masses with Met, Hpg and Aha incorporated proteins might be due to cleavage of 8 residues. We generally could observe these peaks with almost all of the samples of GFPhs-r5M variants. The GFPhs-r5M containing Hpg and Aha showed the mass shift of −22 and −5 Da respectively, compared to GFPhs-r5M with Met. (TIF) [file pone.0046741.s003.tif]

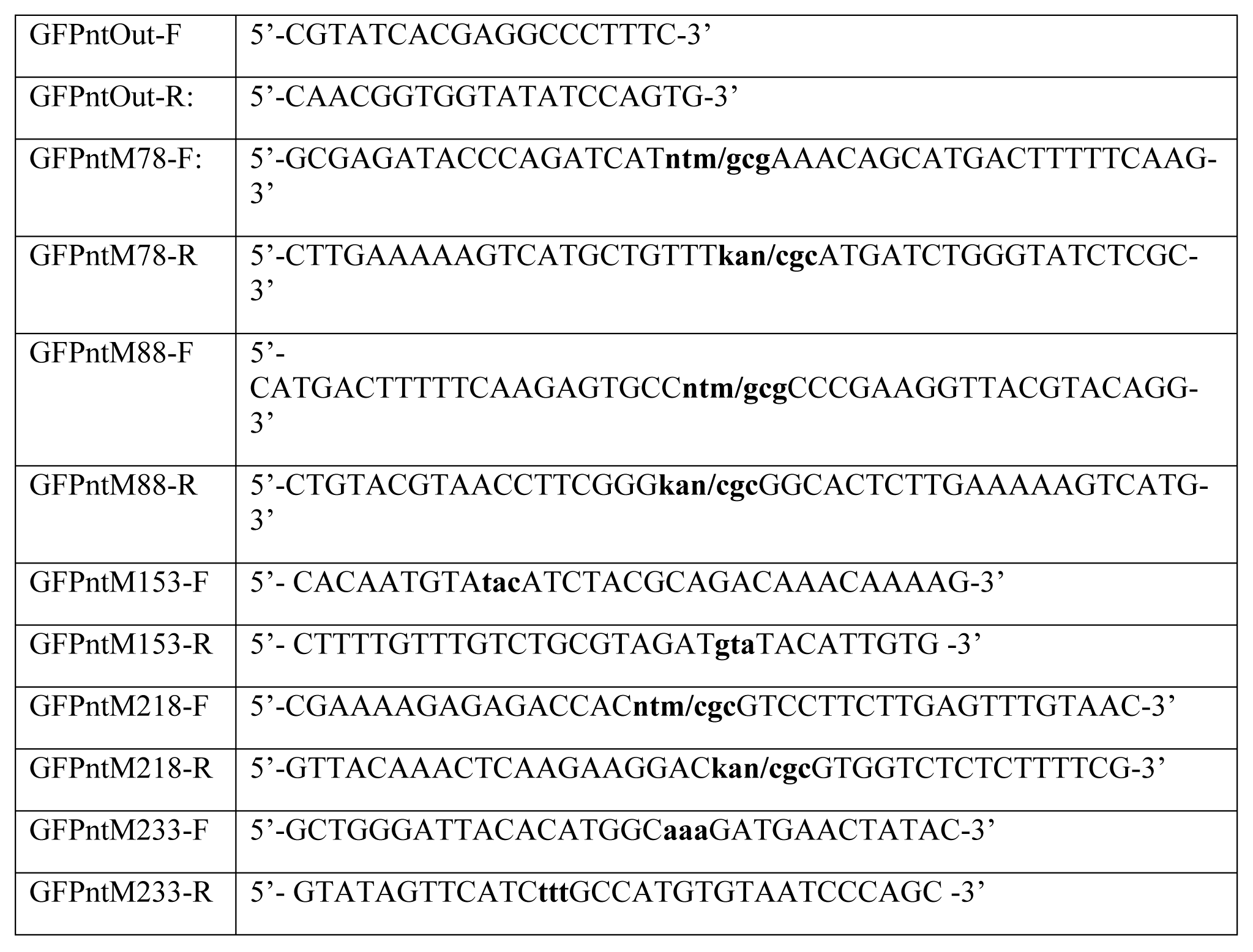

Supplement: Table S1 — Oligonucleotides used for saturation mutagenesis of internal Met-free GFP construction. (TIF) [file pone.0046741.s004.tif]

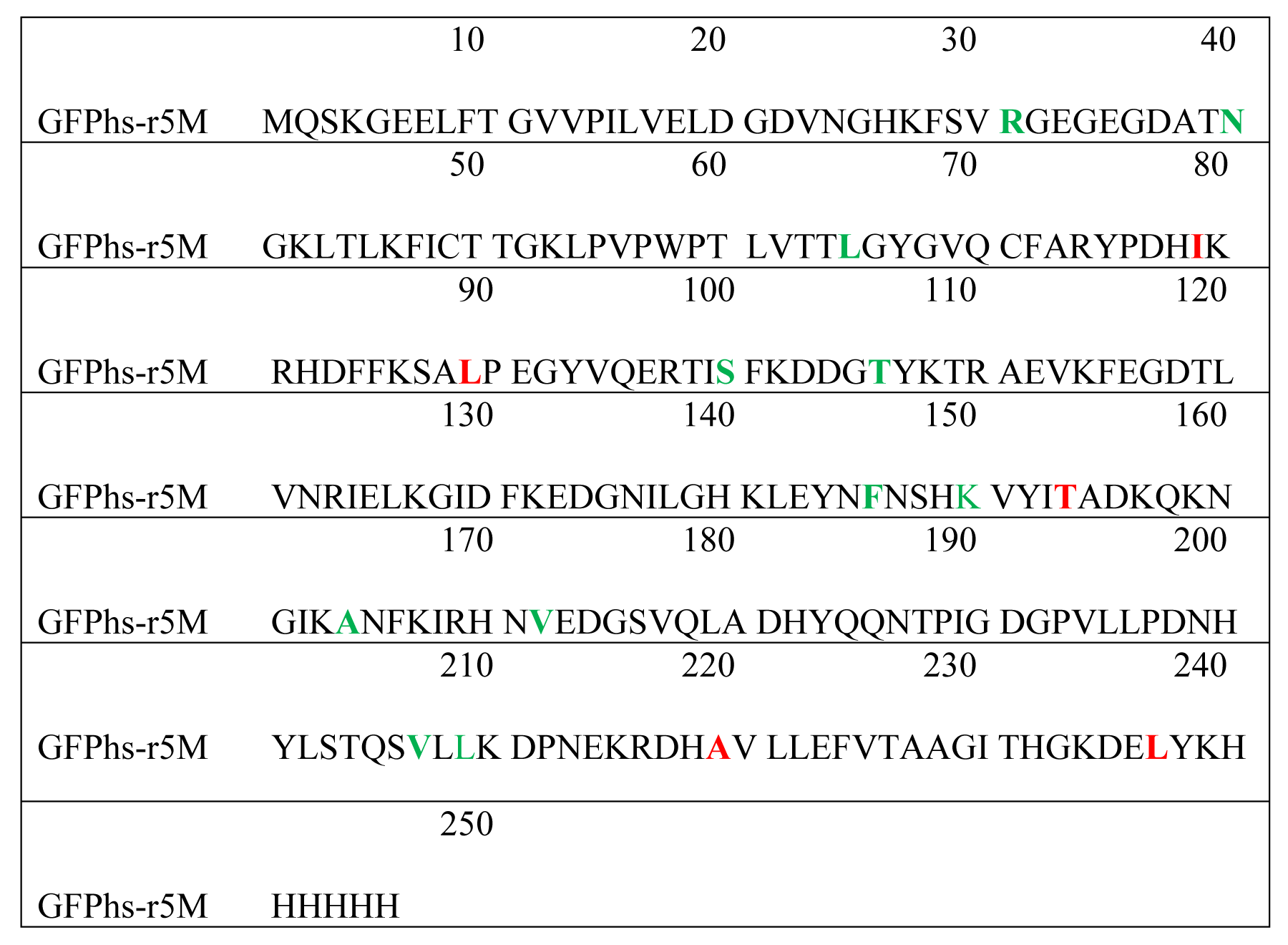

Supplement: Table S2 — Amino acid sequence of the GFPhs-r5M. Red indicates Met replacement mutations, and green indicates the mutations for folding enhancement. The variant expressed as recombinant protein contains a hexahistidine tag sequence in the C-terminus of the protein for Ni-NTA purification. (TIF) [file pone.0046741.s005.tif]
